# Demersal Fish Assemblages and Spatial Diversity Patterns in the Arctic-Atlantic Transition Zone in the Barents Sea

**DOI:** 10.1371/journal.pone.0034924

**Published:** 2012-04-17

**Authors:** Edda Johannesen, Åge S. Høines, Andrey V. Dolgov, Maria Fossheim

**Affiliations:** 1 Ecosystem Processes Research Group, Institute of Marine Research, Bergen, Norway; 2 Pelagic Fish Research Group, Institute of Marine Research, Bergen, Norway; 3 Laboratory of Trophology, Polar Research Institute of Marine Fisheries and Oceanography, Murmansk, Russia; 4 Ecosystem Processes Research Group, Institute of Marine Research, Tromsø, Norway; Texas A&M University-Corpus Christi, United States of America

## Abstract

Direct and indirect effects of global warming are expected to be pronounced and fast in the Arctic, impacting terrestrial, freshwater and marine ecosystems. The Barents Sea is a high latitude shelf Sea and a boundary area between arctic and boreal faunas. These faunas are likely to respond differently to changes in climate. In addition, the Barents Sea is highly impacted by fisheries and other human activities. This strong human presence places great demands on scientific investigation and advisory capacity. In order to identify basic community structures against which future climate related or other human induced changes could be evaluated, we analyzed species composition and diversity of demersal fish in the Barents Sea. We found six main assemblages that were separated along depth and temperature gradients. There are indications that climate driven changes have already taken place, since boreal species were found in large parts of the Barents Sea shelf, including also the northern Arctic area. When modelling diversity as a function of depth and temperature, we found that two of the assemblages in the eastern Barents Sea showed lower diversity than expected from their depth and temperature. This is probably caused by low habitat complexity and the distance to the pool of boreal species in the western Barents Sea. In contrast coastal assemblages in south western Barents Sea and along Novaya Zemlya archipelago in the Eastern Barents Sea can be described as diversity “hotspots”; the South-western area had high density of species, abundance and biomass, and here some species have their northern distribution limit, whereas the Novaya Zemlya area has unique fauna of Arctic, coastal demersal fish. (see Information S1 for abstract in Russian).

## Introduction

**Figure 1:**
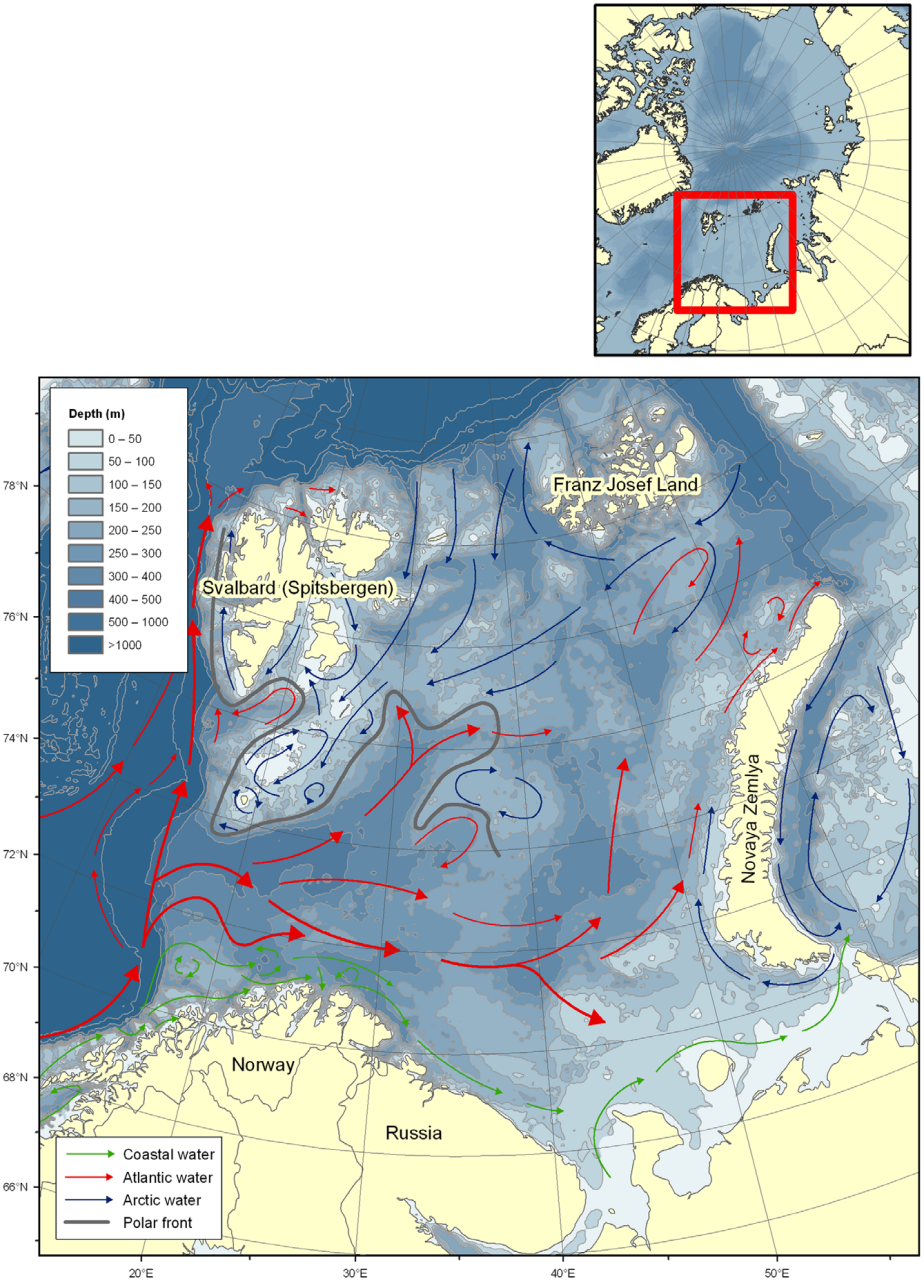
The Barents Sea area with main oceanographic features (after Loeng 1991). Green arrows: coastal water, red arrows: Atlantic inflow and Blue arrows: Arctic water. The position of the polar front (grey) where Arctic and Atlantic water masses meet, is determined by the bottom topography and is well defined in the western Barents Sea.

Direct and indirect effects of global warming are expected to be pronounced and fast in the Arctic, impacting terrestrial, freshwater and marine ecosystems [1]. The Arctic marine ecosystems comprise the deep Arctic Ocean with its surrounding continental shelves and marginal seas, of which the Barents Sea (BS) is the largest (1.6 million km^2^) extending from the shelf break towards the Norwegian Sea in the south (ca. 68°N) to the shelf break in the high Arctic at around 81°N (Figure 1).

The Norwegian pioneer natural historian Michael Sars (1851, cited in [2]) may have been the first to note that the BS is a boundary area between arctic and boreal faunas. The species belonging to different faunas are likely to respond differently to climate variation and trends. Species distributions in the BS largely reflect the oceanographic conditions comprising of Arctic and Atlantic water masses. Outflow of cold, low-salinity Arctic water converges with warmer, saline Atlantic inflow water along the Polar Front (Figure 1, [3]). The position and configuration of the Polar Front is of particular ecological and biogeographical importance and is to a large extent determined by geomorphological features such as deep troughs (>400 meters) and shallow banks (<100 meters). The Polar Front is relatively stable and well defined in the western BS, less so in the eastern BS. In winter, the area north of the Polar Front is covered with ice. Up until recently the north-eastern BS has had permanent ice-cover, but during the last decade the entire shelf sea has been ice free during the summer months [4].

The preference for certain environmental conditions defines species assemblages, i.e. groups of species consistently co-occurring within limited subareas, also within the often large zoogeographical provinces (e.g. [5]). For demersal fish, depth and temperature are apparently the most important habitat variables (e.g. [6],[7]). The BS is a very significant fishing area and most of the commercial fish stocks are monitored regularly. However, more holistic assessments and monitoring of biodiversity and specifically fish assemblages and non-target species are lacking. Such assessments are called for and are now being initiated, however, and baseline descriptions of the fish assemblages form important and necessary foundations for subsequent quantitative studies [8].

More than 200 fish species have been recorded in the BS (e.g. [9], [10]). By comparison 242 marine fish species have been recorded for the whole Arctic region [11]. Few BS fish species have been studied extensively, and in particular many of the Arctic species are poorly described ecologically and even taxonomically (e.g. [11]). Comprehensive faunistic studies in the BS have presented species lists and qualitative classifications of species into zoogeographical and/or ecological groups [12]. [13], [14], [15]. [9], [16], [17]. According to these about ninety percent of the fish species in the BS are benthic or benthopelagic demersal fish. With some notable exceptions (e.g. [12]), the bulk of previous fish research in the BS focused on the 5–10 commercially important target species (e.g. [18], [19]); and accounts of spatial distribution and assemblage structure has only comprised restricted subareas of the BS [20], [21], [22], [23], [24].

Research vessel surveys provide data potentially suitable for comprehensive studies of fish assemblages, not only the species targeted by fisheries. This data base is thus a largely untapped resource of information on the wider fish community and an aim of this paper was to fill a significant knowledge gap by identifying and characterising demersal assemblages based on recent survey data. We consider such studies timely for several reasons: First, species composition and distribution of single species and assemblages within the BS may be affected by the expected alteration in oceanographic features associated with global and regional climate change. A comprehensive account of structure and patterns of distribution of assemblages based on recent data is needed as a baseline against which possible future developments are assessed and monitored. Second, as in other highly impacted marine ecosystems e.g. the North Sea and the Baltic, identifying areas with unique species composition and diversity “hot spots” is of clear relevance to management and the monitoring of direct human impacts (e.g. [25], [7], [26]). The BS is heavily influenced by fishing and it ranks as one of the most important fishing areas of the North Atlantic for a range of boreal fish resources, including the world's largest stock of Atlantic cod (*Gadus morhua*). Development of oil and gas exploration and exploitation is imminent [27]. This strong human presence places great demands on scientific investigation and science-based advisory capacity.

The data used here were collected during the period 2004–2009 on the annual ecosystem survey run jointly by the Institute of Marine Research (IMR), Norway, and the Polar Research Institute of Marine fisheries and Oceanography (PINRO) of the Russian Federation. This survey was run in August-September when the BS has least ice-cover. The study years were the warmest on record extending back to 1900 (∼1°C higher than the long term mean measured in the Kola section in south eastern Barents Sea [28]), and in particular the area of Arctic water in summer (bottom temperatures <0°C) was reduced from 35.8% (average 1970–2003) to 23% (average 2004–2009 [4]). Furthermore, the ice-cover was reduced and extensive northern and eastern areas were ice-free and accessible to the investigations for the first time. The data set from this survey series is the spatially most extensive from the BS, allowing a study of the distribution of fish assemblages across the entire area. No previous analyses considered the spatial variation in fish diversity and assemblages including also species inhabiting the understudied northern BS. Our aim was to create a new baseline by identifying and characterizing the main demersal fish assemblages in the BS in relation to gradients in depths and temperature. From our analysis we identified and characterised six distinct assemblages that were well separated along depth and temperature gradients. These included three Arctic and three Atlantic assemblages. There are indications that climate driven changes have already taken place, since boreal species were found in large parts of the BS shelf, including also the northern Arctic area.

## Results

### Identifying demersal fish assemblages

In total 101 species or species groups were recorded during the survey series (online Information S2). Of these 75 species groups were retained and used further in the cluster analyses after excluding deep (>500 m) and shallow (<50 m) hauls, pelagic species and after merging some taxa with uncertain identity that often were identified only to the family or genus level. The latter were *Gymnelus* sp (dominated by *Gymnelus retrodorsalis*), *Icelus bicornis* and *Icelus spatula* which were pooled as *Icelus* sp., and all liparids treated as *Liparidae*. The liparids were mostly either from the genus *Careproctus*, which may be 2–6 species (or more) with unresolved taxonomic status, or *Liparis fabricii*. The species caught were classified into zoogeographical groups according to [14]; 31% belonged to the group of Arctic or Arcto-boreal species, 60% belonged to Boreal or Mainly Boreal species and 8% belonged to widely distributed or South Boreal species (Information S2).

**Figure 2:**
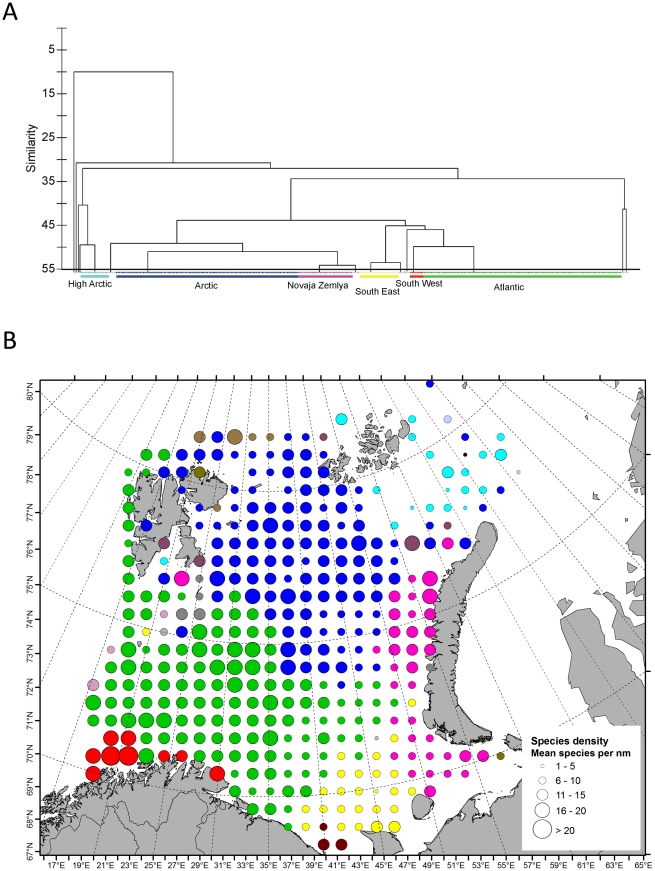
Demersal fish assemblages in the Barents Sea indentified from hierarchical clustering. The main clusters are “South West” (red), “South East” (yellow), “Atlantic” (green), “Arctic” (blue), “High Arctic” (turquoise), and “Novaya Zemlya” (purple). Top: Simplified dendrogram for hierarchical clustering of grid cells showing the six. The full dendrogram is provided in Figure S1. Bottom: Map of the assemblages identified by the cluster analysis. Grid cells belonging to the same cluster are shown with the same color. The size of the circle is proportional to the grid specific species density (the average number of species per nautical mile towed).

The hierarchical cluster analysis of the presence/absence matrix revealed six prominent clusters at 55% similarity (Table 1, Figure 2a, Figure S1). In addition several smaller clusters occurred, consisting of only 1–3 grid cells each. The largest assemblages were an ‘Atlantic’ assemblage in the south and an ‘Arctic’ assemblage north of the Polar Front, comprising 132 and 114 grid cells, respectively (Figure 2b). To the northeast a ‘High Arctic’ assemblage was found around Franz Josef's Land. Along the coasts of Norway and Russia three different coastal clusters occurred; one in the southwest, one in the southeast, and one close to Novaya Zemlya. East of Spitsbergen in the western BS (on the Svalbard bank), single grid cells clustered with each of the three eastern assemblages (“High Arctic”, “Novaya Zemlya” and “South East coastal”). These apparently odd grid cells and the clusters containing only 1–3 grid cells were excluded from further analysis.

**Table 1:** Main assemblages identified from the hierarchical clustering.

	Stations	Grid cells	% Similarity
*Assemblage*			
Atlantic	1368	132	63.5%
Arctic	967	114	65.8%
High Arctic	20	17	61.0%
Novaya Zemlya	186	35	69.8%
Coastal South West	67	10	75.2%
Coastal South East	187	25	66.7%

The number of stations, number of grid cells (each 35 nm by 35 nm) in each assemblage and percent similarity between grid cells in the identified assemblages is provided.

**Table 2:** Pair-wise percentage dissimilarity between the assemblages defined by the cluster analysis.

Assemblage	High Arctic	Arctic	Novaya Zemlya	South East	South West	Atlantic
High Arctic						
Arctic	53.67					
Novaya Zemlya	70.18	48.57				
South East	85.43	59.39	54.73			
South West	92.86	73.15	77.47	59.53		
Atlantic	74.1	50.47	61.48	52.82	50.06	

The boxes encircles the Northern Assemblages (upper left) and Southern Assemblages (lower right).

Globally, there was a significant differences in species composition among the assemblages (p = 0.01). The pairwise tests (Table 2) revealed significant differences in all six main assemblages. The “High Arctic” assemblage differed most from the other assemblages but was most similar to the “Arctic” assemblage (Table 2). The “South West” assemblage was also distinct and was most similar to the “Atlantic” assemblage. The “South West” and” High Arctic” were the most dissimilar assemblages in terms of species composition (Table 2), not unexpected since they were also the most geographically distant (Figure 2b). The “Novaya Zemlya” and “Arctic” assemblage was most similar (dissimilarity = 48.57%). The “Atlantic” and “Arctic” assemblages had a dissimilarity of 50.47%.

### Species composition in the assemblages

The occurrence of all species caught by assemblage is given in Information S2. Four species, cod, Atlantic hookear sculpin, long rough dab and daubed shanny, were ubiquitous and all of these were Mainly Boreal (Information S2). These four species dominated in different assemblages. Long rough dab was among the five most important species (defined as among the five most abundant species of the species with highest occurrence in each assemblage) in all but the “High Arctic” assemblage. Atlantic hookear sculpin was found in >50% of the grid cells in all assemblages and was among the five most important species in both the “Arctic” and the “Atlantic” assemblages, whereas Daubed shanny was among the top five in the “South East” and “Arctic” assemblages. Cod was among the five most important species in the southern assemblages (“Atlantic”, “South East” and “South West”).

The ‘Atlantic’ assemblage was dominated by five species, but only two were found in all 132 grid cells; long rough dab and cod (Table 3). Also thorny skate, Atlantic hookear sculpin, and haddock were found in all but a few grid cells. These five species constituted 44% in terms of numbers and 58 % in terms of biomass (summed across grid cells averages) in this assemblage.

**Table 3:** Demersal fish species dominance given as the five most important species in each assemblages (Figure 2, Table 1).

	Rank (occ.)	Species with highest occurrence	Percentage of total catch by weight	Average catch (kg/nm)	Percentage of total abundance	Average catch (n ind./nm)
Arctic	2	Bigeye sculpin	2	0.8	23	119
Arctic	2	Long rough dab	23	12.6	23	117
Arctic	1	Snailfishes (*Liparidae*)	1	0.7	11	55
Arctic	1	Atlantic hookear sculpin	1	0.4	9	44
Arctic	2	Daubed shanny	<1%	0.1	4	20
Atlantic	1	Long rough dab	11	12.7	17	110
Atlantic	4	Haddock	21	25	15	101
Atlantic	1	Cod	24	28.7	10	64
Atlantic	3	Atlantic hookear sculpin	<1%	93g	2	13
Atlantic	2	Thorny skate	2	1.9	<1%	2
High Arctic	1	Snailfishes (*Liparidae*)	8	0.3	49	37
High Arctic	1	Bigeye sculpin	3	0.1	21	16
High Arctic	2	Greenland halibut	82	3.1	14	11
High Arctic	2	Atlantic poacher	1	29g	3	3
High Arctic	3	*Pale eelpout*	1	22g	3	2
Novaya Zemlya	2	Long rough dab	20	10.5	34	125
Novaya Zemlya	1	Arctic staghorn sculpin	<1%	0.9	11	40
Novaya Zemlya	2	Northern alligatorfish	<1%	55g	6	23
Novaya Zemlya	1	Ribbed sculpin	<1%	0.3	5	19
Novaya Zemlya	2	Atlantic poacher	<1%	78g	4	13
South East	1	Long rough dab	12	25.0	40	263
South East	1	Haddock	46	94.0	38	250
South East	1	Cod	36	72.4	16	107
South East	2	Daubed shanny	<1%	15g	1	4
South East	1	Thorny skate	1	2.0	<1%	2
South West	1	Norway pout	11	34.8	55	1440
South West	1	Haddock	40	132.1	10	273
South West	1	Saithe	18	59.6	2	49
South West	1	Cod	10	33.8	1	37
South West	1	Long rough dab	<1%	1.2	1	31

For each assemblage the species were ranked by occurrence (number of grid cells occupied by the species). The most the five most important species was defined as the five most frequently occurring, if more than five species had the same high occurrence, these were ranked according to their abundance. For the five most important species in each assemblage, the species-specific percentages of the total number of individuals caught and total catch in kg, together with the average number of individuals (n ind./nm) and biomass (kg/nm) (standardised catch per nautical mile towed) is provided.

In the ‘Arctic’ assemblage no species was found in all grid cells, but 6 species groups were found in all but one or two grid cells; bigeye sculpin, long rough dab, Atlantic hookear sculpin, Atlantic poacher, snailfishes (*Liparidae*), and daubed shanny (Table 3). Except for long rough dab, these constituted 70% of the catches in terms of numbers. These are all small species thus contributing only 28% to the weight of the catches. Cod and Greenland halibut were the species that together with long rough dab were most important in terms of biomass in the ‘Arctic’ assemblage.

The ‘High Arctic’ assemblage was dominated by four species, but only two were found in all grid cells; big eye sculpin and snailfishes (Table 3). Atlantic hookear sculpin and Greenland halibut were found in several of the grid cells. The five most common species constituted 90% in terms of numbers and 95 % of the weight of the catches in the ‘High Arctic’ assemblage.

The coastal ‘Novaya Zemlya’ assemblage had two species in all grid cells; the Arctic staghorn sculpin and ribbed sculpin. Additional four species were found in 34 out of the 35 grid cells; northern alligatorfish, Atlantic poacher, daubed shanny and long rough dab (Table 3). The five most abundant species constituted 60% of the catches in numbers and 23 % of the biomass (Table 3). Cod was found in 33 of the 35 grid cells and contributed 55% of the biomass.

The ‘South-East’ coastal assemblage had four species found in all grid cells; long rough dab, cod, haddock and thorny skate. Daubed shanny was found in all but one grid cell (Table 3). These 5 species represented 95 % both of the numbers caught and of the biomass in this assemblage.

In the ‘South-West’ coastal assemblage 9 species were found in all the grid cells. The five most abundant species constituted 69 % of the catches in numbers and 80 % of the catches in biomass.

### Biomass and abundance by assemblage

**Figure 3:**
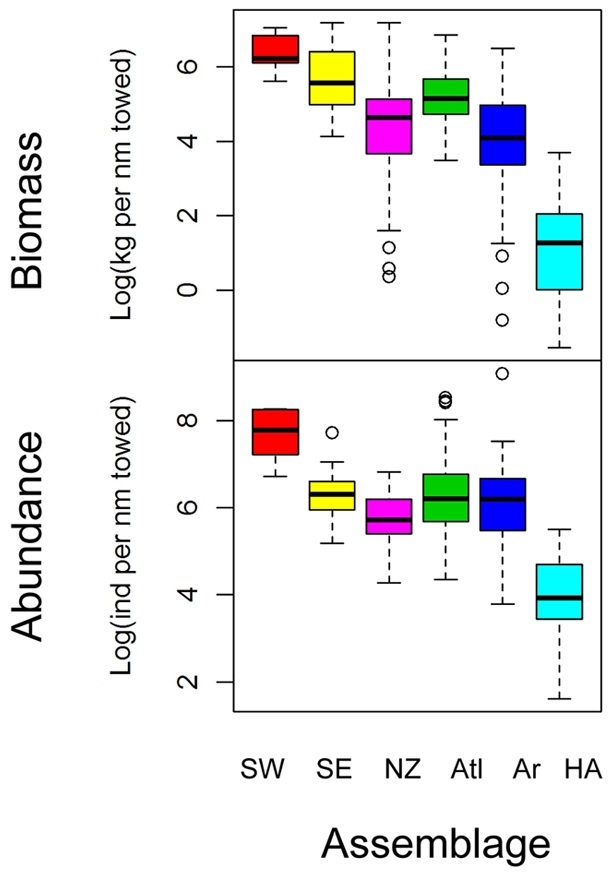
Box plots of grid cell average abundances (bottom) and biomasses (top) by assemblage. The colors represent the different assemblages: “South West” (SW, red), “South East” (SE, yellow), “Novaya Zemlya” (NZ, purple) “Atlantic” (Atl, green), “Arctic” (Ar, blue) and “High Arctic” (HA turquoise).

The average biomass and abundance differed between the assemblages (Figure 3). The highest biomass and abundance was found in the “South West” assemblage and the lowest in the “High Arctic”. The biomass in the “South West” assemblage was significantly higher (p<0.001) than the biomass in all other assemblages except the “South East” assemblage (p = 0.08). The abundance in the “South West” assemblage was significantly higher than the abundance in all the other assemblages (p<0.0001).

### The relationship between species assemblages and depth and temperature gradients

**Figure 4:**
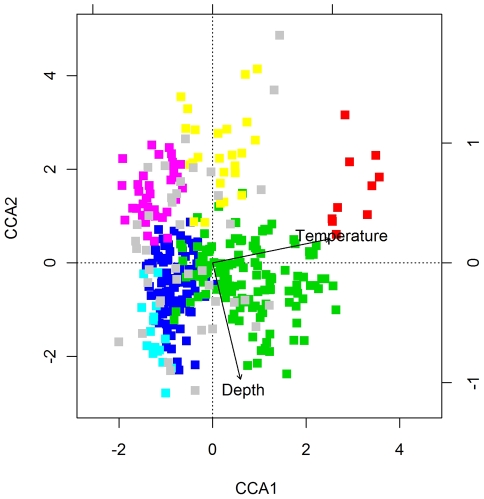
Constrained Correspondence Analysis biplot of axes I and II relating species composition (presence/absence) to environment (bottom temperature and depth). The squares represent grid cells, and their colors represent the different main assemblages: unclassified (grey), “South West” (red), “South East” (yellow), “Atlantic” (green), “Arctic” (blue), “High Arctic” (turquoise), and “Novaya Zemlya” (purple).

**Figure 5:**
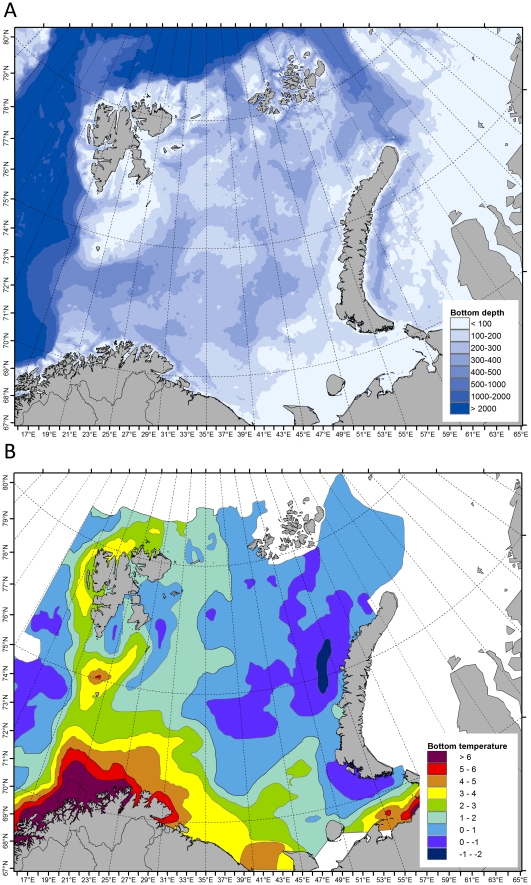
Depth and bottom temperature in the Barents Sea. The depth contours is shown in the top panel (A) and the average bottom temperatures from 2004–2009 is shown at the bottom (B).

To study the relationship between species assemblages and depth and temperature we ran a Constrained Correspondence Analysis with the presence/absence data matrix with grid-specific average bottom temperatures and depths added (Figure 4, Figure 5). The first two constrained ordination axes, accounting for 13.5% (CCA1: 9.2%, CCA2: 4.3%) of the total variation, summarize the spatial component of variation correlated with temperature (∼CCA1), and depth (∼CCA2), respectively (Figure 4).

The assemblages were quite well separated along the depth and temperature gradients. Depth separated the shallow “South East”, “South West” and “Novaya Zemlya” assemblages from the deeper “Atlantic”, “Arctic” and “High Arctic” assemblages. These two groups of shallow and deep assemblages were both separated along a temperature gradient. The “South West” assemblage was the warmest, the “South East” was intermediate and “Novaya Zemlya” was coldest among the shallow, whereas the “Atlantic” was the warmest, the “Arctic” was intermediate and the “High Arctic” was the coldest of the deep assemblages.

### Species diversity by assemblage

**Figure 6:**
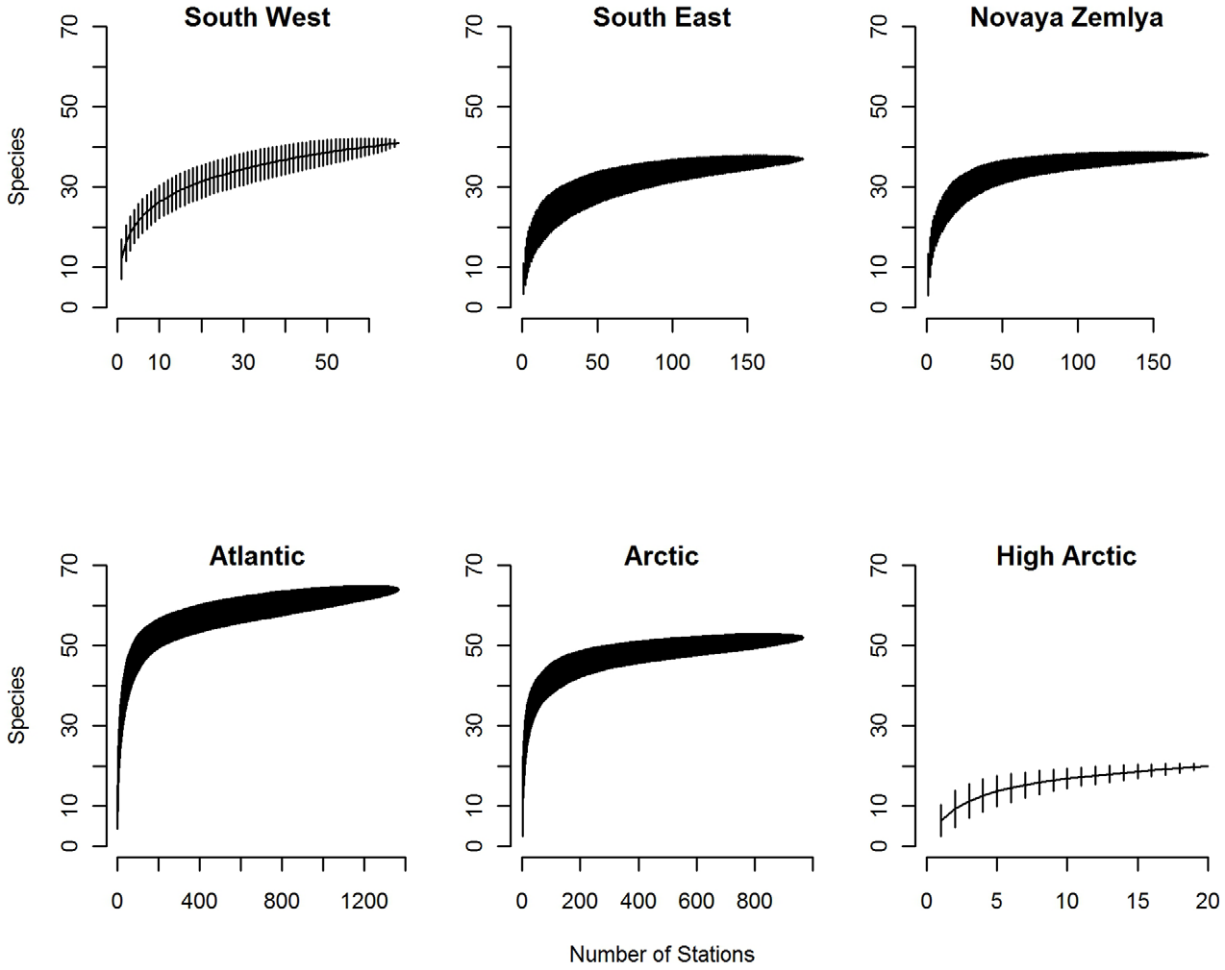
Species accumulation plots for each of the main Barents Sea assemblages. Y-axis: cumulative number of species recorded, x-axis: number of stations sampled.

**Figure 7:**
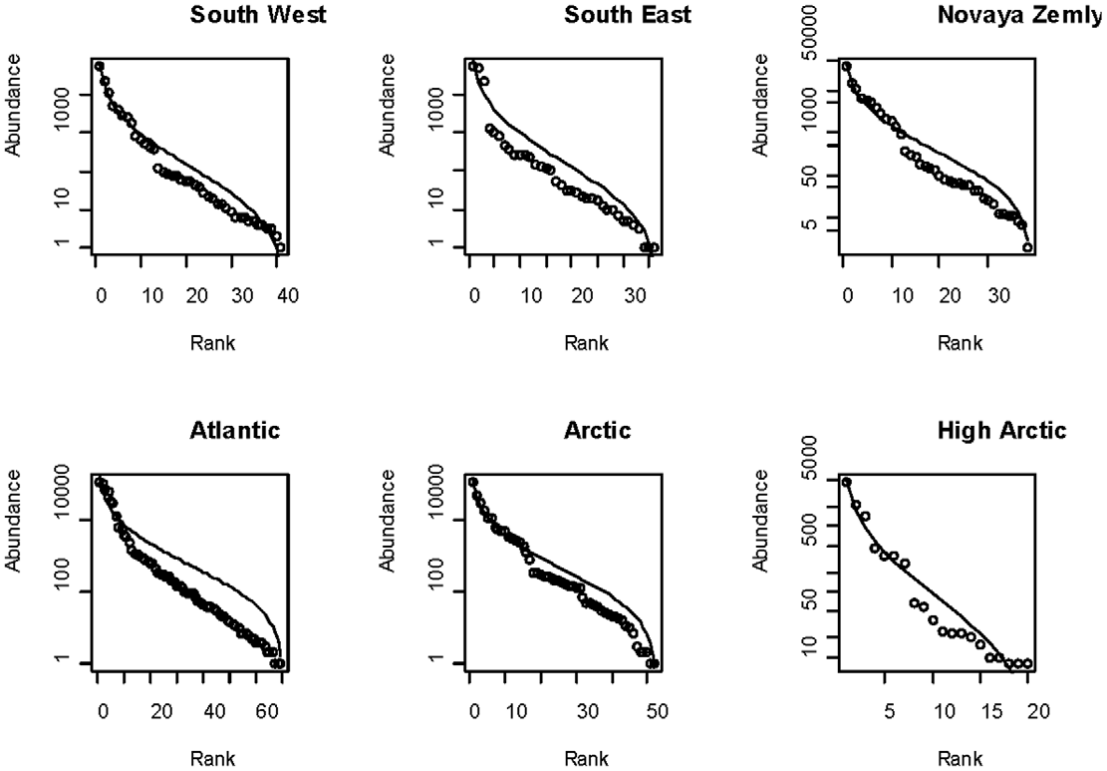
The log abundance species rank plot for each main assemblage. The dots represents the species, the solid line represents the Log normal distribution fitted to the species abundance distribution for each assemblage.

For all six assemblages the species accumulation curves were asymptotic (Figure 6), suggesting that the sampling level was adequate to estimate total species numbers. The two assemblages covering large areas, i.e. the “Arctic” and the “Atlantic” differed in that the warmer “Atlantic” had higher species richness than the colder “Arctic” assemblage (Figure 6), but the difference was rather small. Out of the assemblages covering much smaller area, the “South West” and “High Arctic” differed most in species richness, the asymptotic level being twice as high in the South West than in the High Arctic assemblage (Figure 6). The most common species appeared to show a higher abundance than predicted by the log normal distribution in all communities (Figure 7). In particular, the ”South East” assemblage had a higher dominance of common species and a lower evenness compared to the other assemblages (Figure 7).

**Figure 8:**
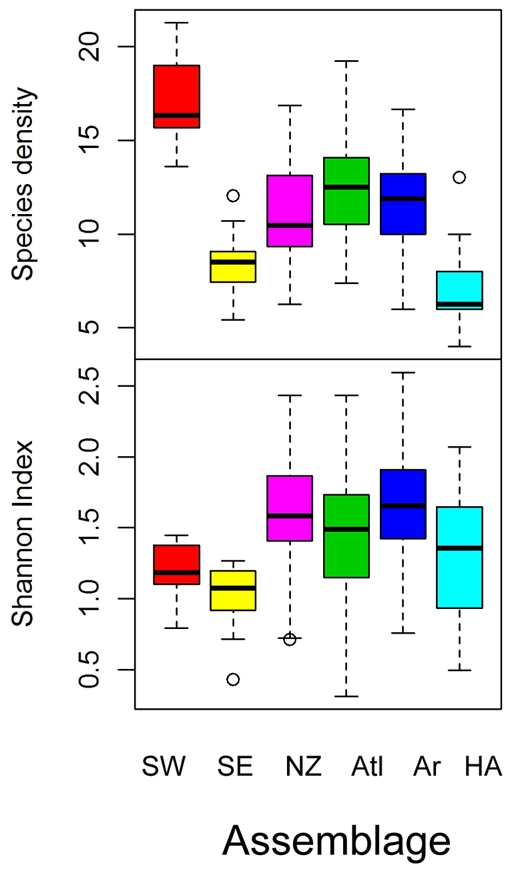
Box plots of grid cell average Shannon index (bottom) and species density (top) by assemblage. The colors represent the different assemblages: South West” (SW, red), “South East” (SE, yellow), “Novaya Zemlya” (NZ, purple) “Atlantic” (Atl, green), “Arctic” (Ar, blue) and “High Arctic” (HA turquoise).

The species density (s) and Shannon index (H′) varied between the assemblages (Figure 8). The species density was highest in the “South West” and lowest in the “High Arctic” assemblage. The species density in the “South West” was significantly higher than in all other assemblages (p<0.0001). The Shannon index was lowest in the “South East”. There was no significant difference between the Shannon index in “South East”, South West” and “High Arctic”, but these were significantly lower than the Shannon index in the “Arctic”, “Atlantic” and “Novaya Zemlya” assemblages (p<0.05).

### Species diversity according to depth and temperature

**Figure 9:**
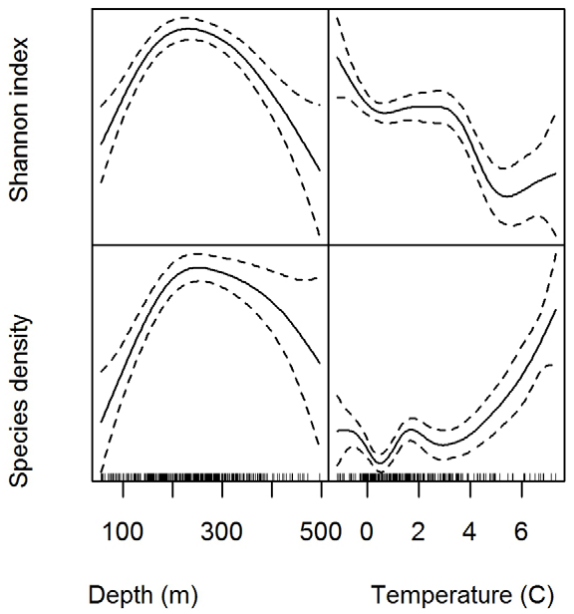
Shannon index (top) and Species density (bottom) as function of depth (left) and temperature (right). The relationships were fitted with generalized additive models, with grid cells as the statistical unit (n = 374).

The grid cell specific species density and Shannon index varied significantly with depth and temperature (p<0.0001), explaining 32.1 and 34.5% of the deviance in species density and Shannon index, respectively. Shannon index declined and species density increased non-linearly with temperature (Figure 9). Both Shannon Index and species density was highest at intermediate depths, but the pattern was most pronounced for the Shannon index (Figure 9). There was however, strong spatial variation in the residuals (spline interaction term between latitude and longitude, p<0.0001, both Shannon index and species density).

**Figure 10:**
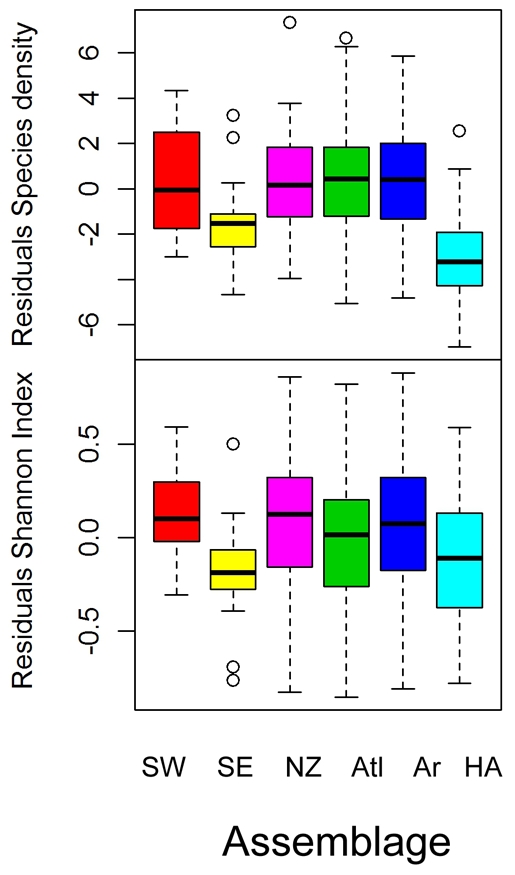
Box plots of residuals from modeling diversity as function of temperature and depth by assemblage. Residuals from fitting the Shannon index is shown at the bottom and residuals from fitting species density are shown at the top. The colors represent the different assemblages: “South West” (SW, red), “South East” (SE, yellow), “Novaya Zemlya” (NZ, purple) “Atlantic” (Atl, green), “Arctic” (Ar, blue) and “High Arctic” (HA turquoise).

The residuals differed significantly between the assemblages (Figure 10). The residuals from the depth and temperature analysis for species density were significantly lower both for the “South East” and “High Arctic” assemblages (p<0.05). The residuals from the depth and temperature analysis for the Shannon index was significantly lower in the “South East” assemblage compared to the others (p = 0.03). This means that there was significantly lower diversity in these assemblages than predicted from their temperature and depth estimated at the scale of the whole of the BS shelf.

## Discussion

In this medium-large scaled (∼1.6 million km^2^) and coarse grained (∼35 by 35 nm sampling units) study we identified assemblages that all had reasonable levels of internal similarity (>60%) and that were reasonably distinct (all pairwise dissimilarities >48.5%). These levels of similarity/dissimilarity between major clusters are in the same range as commonly found in other investigations on marine assemblages (e.g. [29], [30], [31], [32]). The spatial distribution of the main assemblages was quite robust even when analysing species matrices of abundance and biomass data and when doing the analysis by separate years (Å. Høines unpublished results). Consequently pooling the data for all years did not mask or alter the main conclusions. We used the distance metric and linkage most commonly used for these type of studies (e.g. [33], [34], [35], [36]), and did not evaluate how these choices might influence our results. We did however, analyse also data on abundance and biomass. These analyses did result in one or two additional noticeable assemblages along the polar front where the abundance and biomass of the most common species were highest, compared to the analysis on presence absence.

Species density, often taken to be identical with species richness [37], may be a sampling artefact of overall individual density, since the probability of sampling individuals of rare species increase with overall density, given the same sampling effort. This effect might contribute to some of our results, e.g. the “South-West” assemblage had high species densities and overall abundances, whereas the “High Arctic” had low species density and abundances. However, our species accumulation plots show that the species pool was higher in “South West” than in the “High Arctic”, a similar sized area; therefore higher species densities reflect overall higher species richness in addition to higher abundances.

We used catch data from demersal trawl; a highly selective sampling gear. Different species of fish behave differently ahead of the trawl gear (discussed in [38]). The main issue that could bias the cluster analysis is if there is spatial variation in the species specific catchability. To estimate absolute species densities and actual species composition the catchability of the different fishes under different conditions should have been evaluated [39]. This would have required targeted studies and was outside the scope of the present study.

Our identified assemblages can be used as baseline when studying future changes. Like in [23] studying a restricted area in the western BS, we found a discontinuity between the Atlantic and Arctic part of the BS. The northern, Arctic part is ice covered in winter, whereas the Boreal/Atlantic part is more influence by Atlantic inflow. The Northern “High Arctic”, “Novaya Zemlya” and “Arctic” assemblages had small catch rates, especially in terms of biomass, and small-bodied species dominated in terms of occurrence. In contrast, the southern group of assemblages, the “Atlantic”, “South East” and “South West” had high catch rates in kg and higher occurrence of large bodied species like cod and haddock.

Still, the spatially extensive “Atlantic” and “Arctic” assemblages were not very distinct in terms of species composition and diversity, probably partly attributable to the intrusion of seasonally migrating boreal species into the northern part of the BS at this time of year (August-September). Our finding that boreal species occurred in large parts of the BS, including also the Arctic north of the polar front, may be an indication that distributional changes due to the recent warming trend has already taken place, probably caused by extended seasonal migrations in the exceptionally warm study years [28]. In particular, the northern part of the BS was warmer than normal. In this area Arctic water was heated by sub-ducted Atlantic water following the western slope of the BS Shelf, north along the western part of Spitsbergen and entering from the north between the Svalbard/Spitsbergen archipelago and Franz Josef Land [4].

Like in previous warm periods in the BS (e.g. the 1930's, [40]), new southern species are expected to extend into the BS and change the species composition and increase species richness in the Southern BS. Many of the species that are expected to occur already co-exist in the North Sea with the boreal species present in the BS. Common boreal species already present in the BS will probably extend further into the northern BS, and our results suggest that this is already taking place. The boreal species are constrained by their migration potential, but if climate change induces changes in the spawning areas toward the north these spatial constraints are relieved and major changes in spatial distribution and species composition of the assemblages could be expected.

A relevant question is whether changes due to climate variability and trends can be detected in an area such as the BS where there is a strong influence of fishing, especially on boreal species. Fishing, while mainly targeting certain species, may have a structuring effect on assemblages. The southern BS is similar both in fish species composition and fishing effort to several other North Atlantic shelf ecosystems like the North Sea [41]. In general, fish diversity and fish community structure in these systems have been studied much more extensively than the BS communities. In the North Sea, spatial variation in hydrographical regimes determines the structure of demersal fish community and spatial structure of the fish community was much stronger than temporal variation across a period of 18 years [31]. Similar results have been found in Iceland [42]. However, over a longer time period (71 years) the species composition of demersal fish in the North Sea has changed [43]. This change was mostly induced by fishing since the proportion of fish species that were vulnerable to fishing decreased (e.g. slow growing species with late maturation).Size distributions have often been used to assess changes in fish communities (e.g. [44]) and this is of special relevance when assessing the impact of fishing since fishing removes large-bodied species and individuals [45]. The Arctic fish species in the BS is mostly small bodied; therefore the identity of the species and the species composition is more relevant than the size distribution *per se* when evaluating the impact of climate in relation to fishing on the BS fish community. Furthermore, the spatial variation in fishing pressure has to be taken into account, up to date, the fishing activity in the Northern BS is limited.

Solar radiation/ energy declines with depth and latitude and increases the productivity [46], [47]. Sea temperature is often used as a proxy for solar radiation, energy and productivity in marine systems and therefore servers ti explain the latitudinal gradient in species richness in demersal fish and other marine taxa (e.g. [48], [49], [50]). The effect of depth on species richness in marine ecosystems is equivalent to the effect of altitude in terrestrial systems. Two general patterns are often found: 1) either a decline in species richness with depth/altitude consistent with a drop in energy/productivity (e.g. [51], [52]), or 2) a peak in richness at intermediate depths/altitudes [48], [53]. This latter relationship is explained in terms of the mid-domain effect which is caused by species ranges randomly arranged in a limited domain, will produce a dome shaped relationship between e.g. depth and species richness [54]. For the other aspect of diversity, evenness the empirical relationships with energy, production and depth/altitude and latitude, are less consistent (e.g. [47]).

At the scale of the whole BS, we found a non linear increase in species density with temperature and a dome shaped relationship with depth. The Shannon index, taking both the evenness and species richness into account, had a similar dome shape relationship with depth as species density, but the relationship with temperature was negative. This can be attributed to a decrease in evenness with temperature as judged from the assemblage specific species rank abundance plots.

When accounting for the large scale relationships between diversity, depth and temperature for the whole BS, we found that the species densities and Shannon index in the “South East” assemblage was lower than expected. There are several possible explanations. There is a fishery by demersal trawl mainly for flatfish in the South Eastern part of the BS and demersal trawling can have negative impact on both targeted species and certain vulnerable species taken as by-catch (e.g. [55]). There is, however, area-based data on fishing activity by Norwegian and Russian vessels, and although the data are not directly comparable, it is known that the fishing activities are higher in other parts of the southern BS. Therefore, fishing activity alone is most likely not responsible. Then there is a potential effect of habitat complexity, which might increase fish diversity (e.g. [56]). The area covered by the “South East” assemblage is flat and rather homogenous, and this might contribute to the low diversity. Finally, the “South East” assemblage is far from the entrance of the BS to the Atlantic Ocean, i.e. far from the pool of Atlantic/boreal species. A decline in the number of species from the BS and eastwards to the White Sea and the Kara and Laptev Seas was also found in [57]. Decline in species richness from west to east attributable to distance from the Atlantic Ocean, although at a larger scale, has been found in the Mediterranean (e.g. [58]). The “High Arctic” assemblage is rather similar to fish community in the species poor Kara Sea [59], [60]. In the “High Arctic” assemblage, the lower than expected species density cannot be attributable to fishing, or habitat homogeneity, but the distance to source of Atlantic species might be important [53]. Furthermore, the strong seasonality in this northern area including long periods with ice cover limiting annual productivity might be just as important as the temperature measured during the survet in determining the low species density and species richness (e.g. [61], [62]).

The “South-East” and “High-Arctic” assemblages had low species densities compared to their depth and temperature, whereas the “South-West” and “Novaya Zemlya” can be considered as diversity “hot spots”. The “South-west” assemblage had high species richness compared to the relatively small area it encompassed, and the species density was high. The Shannon index and evenness was low however, since some species was very abundant (e.g. the Norway pout). This area is the distribution limit of several more southerly coastal boreal fish species [63] and the area is very rich in sponges and other benthic invertebrates creating complex three-dimensional habitats (L. Lindal Jørgensen personal communication). The “”Novaya Zemlya” assemblages had, even though the bottom temperatures were the lowest measured during the study, relatively high species density and species richness (e.g. compared to the “South East” assemblage covering a similar sized area) as well as relatively high Shannon index and evenness (as judged from the species rank abundance plot). This area has the highest occurrence of Arctic coastal demersal fishes in the BS, some of which are only found in this part of the BS [64]. These two coastal assemblages thus exemplify the boreal and Arctic faunas characterizing the Barents Sea [2].

In conclusion, we identified and characterized a basic structure of six assemblages and since this is the first study with data available for the entire BS, these can serve as important references when evaluating variation and trends e.g. induced by factors such as climate or direct human impacts (e.g. [42], [65])

## Materials and Methods

### Sampling and input data

The data used were collected on the Joint IMR PINRO ecosystem survey run annually in August and September since 2004 (e.g. [66]). Fish were sampled with a Campelen 1800 bottom trawl towed on double warps [67]. The mesh size was 80 mm (stretched) in the front and 16–22 mm in the codend, allowing the capture and retention of small-sized fish. The trawl configuration and bottom contact was monitored remotely by Scanmar trawl sensors. The horizontal opening was 17 m, and the vertical opening 4–5 m. A rockhopper ground gear was used throughout. The standard distance between bottom trawl hauls was 35 nautical miles (35×1852 m). However, in certain areas where additional investigations were carried out, sampling density was higher. The standard procedure was to tow 15 minutes after the trawl had made contact with the bottom, but the tow duration actually ranged between 5 minutes and 1 hour, and 25% of the tows deviated more than ±2 minutes from the standard towing time. Towing speed was 3 knots, equivalent to a towing distance of 0.75 nautical miles (ca. 1400 m) in a 15 min tow.

Temperature was measured with a conductivity, temperature and depth (CTD) profiler at every sampling site, except in areas with a denser bottom station grid, here a CTD was run at every second bottom trawl station.

In the study period (2004–2009), 3282 bottom trawl hauls were made. Of these 262 were carried out opportunistically specifically to identify and sample echo sounder records of sound-scattering organisms, and these were excluded from our analyses. We also excluded 68 hauls due to technical problems with the trawl gear or operation. Since we were studying demersal fish within the BS proper, i.e. on the continental shelf but not in sublittoral coastal waters, we excluded catches from areas deeper than 500 m (217 hauls) and shallower than 50 m (27 hauls). The dataset analysed thus comprised 2707 valid hauls.

Fish catches were immediately sorted to lowest possible taxonomic level, preferably species. For each species, the wet ungutted catch weight and the number of individuals in the catch was recorded. Pre-analysis data quality checking involved critically examining records. Species not previously reported from the BS were only retained if the specimens (or a photograph of it) had been identified by taxonomists. Some obvious misidentifications occurred of species very unlikely to inhabit the BS were excluded. The numbers of excluded records were low, i.e. about 100 out of more than 35,000 records. More details on the fish records are provided in the BS Fish Atlas resulting from the same survey data [64]. The catches, i.e. weights and numbers per tow, were standardized by dividing catches by the towing distance in nautical miles prior to analysis.

[14] classified 163 fish species recorded in the BS into seven zoogeographical groups (Arctic, Mainly Arctic, Arcto-boreal, Mainly Boreal, Boreal, South Boreal and widely distributed). We classified the species in our data by these categories (Information S2). The species were also categorised into demersal and pelagic species following [14]. Catches of pelagic species in these hauls were excluded (Information S2).

The Shannon index (H′) of diversity was calculated for each tow as:
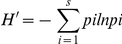
Where S in the total number of species per haul, p_i_ is the proportion of abundance of species *i* in the haul of the total abundance summed across all species caught in the haul.

The data were gridded into a 35 nm by 35 nm grid with 374 grid cells based on the survey design. We calculated grid cell averages of: the standardized number of individuals by species per haul, total number of individuals summed across species per haul, the total biomass in kg summed across species per haul, the species density (that is the number of species per haul),and the Shannon index.

### Statistical analyses

To identify assemblages of fish from co-occurrence hierarchical cluster analysis was applied [35], [68] using PRIMER (Plymouth Routines in Multivariate Ecological Research) for Windows version 6.1.6 (2006). All biotic grid cell similarity matrices used in these analyses were constructed using the Bray-Curtis similarity index [33]. Average linkage was used.

Initially, the hierarchical cluster analyses were run on the gridded species matrix, for presence/absence–, abundance (individuals per nm towed)–and biomass data (kg per nm towed). The abundance–and biomass analyses can often be over-dominated by a small number of highly abundant species, which then fail to reflect similarity of overall community composition [35], [69] and the analyses using the abundance or biomass matrix showed patterns that were strongly influenced by the most common species. Reduction of the data to simple presence/absence values gives rare species equal weight as common ones. Further, due to the undefined uncertainty in the sampling and highly skewed distributions of the species biomass and abundance data, a reduction of the data matrix to simple presence or absence of each species are justified and the final clustering on presence/absence data was presented here.

To test for global differences and pairwise comparisons between the identified species assemblages the Analyses of Similarities (ANOSIM) routine implemented in Primer v6 [68] was used. In all the analyses 999 permutations were used.

To estimate species richness by assemblage, species accumulation plots were obtained by using the R package vegan [70] on station level presence-absence data.

The species abundance distributions were obtained for each assemblage on the gridded standardized abundance data using the package *vegan* [70] was used.

To relate the structural variation in the demersal fish communities to gradients in depth and temperature Canonical Correspondence Analysis (CCA) was used (R package *vegan*, [70]).

To relate the Shannon index and species density to temperatureand depth we used generalised additive models (gams) allowing for non linear relationships (R package *mgcv* [71]).

## Supporting Information

Information S1 **Abstract in Russian**
(DOC)

Information S2 **List of fish species recorded at the ecosystem survey in the Barents Sea 2004–2009.** The species were caught in demersal trawl surveys during the summers 2004–2009 in the Barents Sea. The zoogeographical affinities following [14]: Arctic (A), Mainly Arctic (MA), Arcto-boreal (AB), Mainly boreal (MB), Boreal (B), South Boreal (SB) and Widely Distributed (WD) is shown for each species. Species excluded from the analyses (Excl.) are marked with either **p** (pelagic), **d** (deep>500,) or **s** (shallow<50 m). Notes on identification for certain groups are given below the table. The species are listed together with their frequency of occurrence in the species assemblages resulting from the hierarchical clustering (see text and Fig. 2). The frequency of occurrence is expressed as number of grid cells with records of the species as a proportion of the total number of grid cell in each assemblage. The names of the assemblages are abbreviated: South West: SW, Atlantic: Atl, South East: SE, Novaya Zemlya: NZ, Arctic: Ar, High Arctic: HA.
(DOC)

Figure S1 **The full dendrogram from the hierarchical clustering on the grid cells (n = 374).** The cut-off line at 55% similarity used to determine the clusters is shown in red. The main assemblages discussed in the text are identified by colored lines at the bottom of the dendrogram.
(TIF)
