# Supplementary material for: Demersal Fish Assemblages and Spatial Diversity Patterns in the Arctic-Atlantic Transition Zone in the Barents Sea
Source: PLoS One. 2012 Apr 17;7(4):e34924. doi: 10.1371/journal.pone.0034924 (PMC3328492; doi:10.1371/journal.pone.0034924)
Supplement: Information S1 — Abstract in Russian (DOC) [file pone.0034924.s001.doc]

Реферат

Прямое и косвенное влияние глобального потепления в Арктике наиболее ярко выражено и происходит довольно быстро, оказывая воздействие на наземные, пресноводные и морские экосистемы. Баренцево море представляет собой высокоширотное шельфовое море, где проходит граница между арктической и бореальной фауной. Эти фаунистические комплексы по-разному реагируют на изменения климата. Кроме того, на Баренцево море значительное влияние оказывает промысел и другие виды хозяйственной деятельности. Такое сильное антропогенное воздействие предъявляет высокие требования к научным исследованиям и их возможности разрабатывать рекомендации. Для того чтобы выявить базовую структуру сообществ, которая могла бы быть использована в качестве реперной точки при изучении воздействия климата, мы проанализировали видовой состав и видовое разнообразие донных рыб в Баренцевом море. Были выявлены шесть основных типов ихтиоценов, которые различались глубиной и температурой воды. Были получены доказательства того, что климатические изменения уже привели к изменениям в сообществе рыб, поскольку бореальные виды рыб были обнаружены во многих частях шельфа Баренцева моря, включая его северные арктические районы. При моделировании видового разнообразия как производного от глубины и температуры воды, было выявлено, что для двух типов ихтиоценов в восточной части Баренцева моря характерны более низкое разнообразие, чем ожидалось при соответствующих глубинах и температурах воды. Вероятно, это было обусловлено низким разнообразием условий обитания и значительной удаленностью от районов распределения бореальных видов в западной части моря. В противоположность этому, прибрежные ихтиоцены в юго-западной части моря и вдоль побережья Новой Земли в восточной части моря характеризуются высоким разнообразием; в юго-западном районе отмечалась высокие видовая насыщенность, численность и биомасса видов, в этом же районе находилась северная граница ареала ряда тепловодных видов, в то время как в районе Новой Земли отмечалась уникальная ихтиофауна арктических прибрежных донных видов рыб.
