# Supplementary material for: Demersal Fish Assemblages and Spatial Diversity Patterns in the Arctic-Atlantic Transition Zone in the Barents Sea
Source: PLoS One. 2012 Apr 17;7(4):e34924. doi: 10.1371/journal.pone.0034924 (PMC3328492; doi:10.1371/journal.pone.0034924)
Supplement: Information S2 — List of fish species recorded at the ecosystem survey in the Barents Sea 2004–2009. The species were caught in demersal trawl surveys during the summers 2004–2009 in the Barents Sea. The zoogeographical affinities following [14]: Arctic (A), Mainly Arctic (MA), Arcto-boreal (AB), Mainly boreal (MB), Boreal (B), South Boreal (SB) and Widely Distributed (WD) is shown for each species. Species excluded from the analyses (Excl.) are marked with either p (pelagic), d (deep>500,) or s (shallow<50 m). Notes on identification for certain groups are given below the table. The species are listed together with their frequency of occurrence in the species assemblages resulting from the hierarchical clustering (see text and Fig. 2). The frequency of occurrence is expressed as number of grid cells with records of the species as a proportion of the total number of grid cell in each assemblage. The names of the assemblages are abbreviated: South West: SW, Atlantic: Atl, South East: SE, Novaya Zemlya: NZ, Arctic: Ar, High Arctic: HA. (DOC) [file pone.0034924.s002.doc]

List of fish species recorded. The species were caught in demersal trawl surveys during the summers 2004-2009 in the Barents Sea. The zoogeographical affinities following [14]: Arctic (A), Mainly Arctic (MA), Arcto-boreal (AB), Mainly boreal (MB), Boreal (B), South Boreal (SB) and Widely Distributed (WD) is shown for each species. Species excluded from the analyses (Excl.) are marked with either **p** (pelagic), **d** (deep>500,) or **s** (shallow<50m). Notes on identification for certain groups are given below the table. The species are listed together with their frequency of occurrence in the species assemblages resulting from the hierarchical clustering (see text and Fig, 2). The frequency of occurrence is expressed as number of grid cells with records of the species as a proportion of the total number of grid cell in each assemblage. The names of the assemblages are abbreviated: South West: SW, Atlantic: Atl, South East: SE, Novaya Zemlya: NZ, Arctic: Ar, High Arctic: HA.

| Common name | Species | Zoogr. | | Excl. | | Occurrence by assemblage | | | | | |
| --- | --- | --- | --- | --- | --- | --- | --- | --- | --- | --- | --- |
|  |  |  |  | |  | SW | Atl | SE | NZ | Ar | HA |
| Arctic Lamprey | *Lethenteron camtschaticum* | MB |  | | 0 | 0 | 0 | 0.04 | 0 | 0 | 0 |
| Velvet belly | *Etmopterus spinax* | WD |  | |  | 0.11 | 0 | 0 | 0 | 0 | 0 |
| Greenland shark | *Somniosus microcephalus* | MB |  | | p |  |  |  |  |  |  |
| Spinetail ray | *Bathyraja spinicauda* | MB |  | |  | 0.11 | 0.14 | 0 | 0 | 0.01 | 0 |
| Arctic skate | *Amblyraja hyperborea* | A |  | |  | 0 | 0.19 | 0.04 | 0.19 | 0.30 | 0.11 |
| Thorny skate | *Amblyraja radiata* | MB |  | |  | 0.56 | 0.97 | 1 | 0.22 | 0.40 | 0 |
| Sailray | *Dipturus linteus* | B |  | |  | 0.11 | 0 | 0 | 0 | 0 | 0 |
| Round ray | *Rajella fyllae* | MB |  | |  | 0.78 | 0.33 | 0 | 0 | 0 | 0 |
| Rabbit fish | *Chimaera monstrosa* | B |  | |  | 0.33 | 0.02 | 0 | 0 | 0 | 0 |
|  | *Diastobranchus capensis* |  |  | | d |  |  |  |  |  |  |
| Herring | *Clupea harengus* | MB |  | | p |  |  |  |  |  |  |
| Pacific herring | *Clupea pallasii suworowi* |  |  | | p |  |  |  |  |  |  |
| Greater argentine | *Argentina silus* | B |  | | p |  |  |  |  |  |  |
| Large-eyed argentine | *Nansenia groenlandica* | SB |  | | d |  |  |  |  |  |  |
| Capelin | *Mallotus villosus* | MB |  | | p |  |  |  |  |  |  |
| Smelt | *Osmerus eperlanus* | B |  | | s |  |  |  |  |  |  |
| Atlantic salmon | *Salmo salar* | MB |  | | p |  |  |  |  |  |  |
| Pearlside | *Maurolicus muelleri* | B |  | | p |  |  |  |  |  |  |
| Ribbon barracudina | *Arctozenus risso* | WD |  | | p |  |  |  |  |  |  |
|  | *Myctophidae^1^* |  |  | | p |  |  |  |  |  |  |
| Rough-head grenadier | *Macrourus berglax* | B |  | |  | 0.11 | 0.11 | 0 | 0 | 0 | 0 |
| Arctic cod | *Arctogadus glacialis* | A |  | |  |  |  |  |  |  |  |
| Polar cod | *Boreogadus saida* | A |  | | p |  |  |  |  |  |  |
|  | *Eleginus nawaga* | A |  | | s |  |  |  |  |  |  |
| Silvery pout | *Gadiculus argenteus* | SB |  | | p |  |  |  |  |  |  |
| Atlantic cod | *Gadus morhua* | MB |  | |  | 1 | 1 | 1 | 0.92 | 0.81 | 0.05 |
| Haddock | *Melanogrammus aeglefinus* | MB |  | |  | 1 | 0.94 | 1 | 0.39 | 0.41 | 0 |
| Whiting | *Merlangius merlangus* | SB |  | |  | 0.33 | 0.03 | 0 | 0 | 0 | 0 |
| Blue whiting | *Micromesistius poutassou* | MB |  | |  | 0.89 | 0.61 | 0 | 0 | 0.11 | 0 |
| Pollack | *Pollachius pollachius* | B |  | |  | 0.22 | 0 | 0 | 0 | 0 | 0 |
| Saithe | *Pollachius virens* | MB |  | |  | 1 | 0.42 | 0.31 | 0.06 | 0.05 | 0 |
| Norway pout | *Trisopterus esmarkii* | B |  | |  | 1 | 0.70 | 0 | 0 | 0.08 | 0 |
| Tusk | *Brosme brosme* | MB |  | |  | 1 | 0.18 | 0 | 0 | 0 | 0 |
| Fourbeard rockling | *Enchelyopus cimbrius* | B |  | |  | 0.11 | 0.16 | 0 | 0 | 0 | 0 |
| Arctic rockling | *Gaidropsarus argentatus* | A |  | |  | 0.11 | 0.05 | 0 | 0 | 0.03 | 0 |
| Ling | *Molva molva* | B |  | |  | 0.33 | 0.02 | 0 | 0 | 0 | 0 |
| Greater forkbeard | *Phycis blennoides* | SB |  | |  | 0.11 | 0.01 | 0 | 0 | 0 | 0 |
| European hake | *Merluccius merluccius* | SB |  | |  | 0 | 0.01 | 0 | 0 | 0 | 0 |
| Anglerfish | *Lophius piscatorius* | SB |  | |  | 0.11 | 0.01 | 0 | 0 | 0 | 0 |
| Three-spined stickleback | *Gasterosteus aculeatus* | MB |  | |  | 0 | 0.18 | 0.23 | 0 | 0 | 0 |
| Snake pipefish | *Entelurus aequoreus* | B |  | | p |  |  |  |  |  |  |
| Deepwater redfish | *Sebastes mentella* | MB |  | |  | 0.67 | 0.89 | 0 | 0 | 0.73 | 0.32 |
| Golden Redfish | *Sebastes norvegicus* | MB |  | |  | 0.89 | 0.65 | 0.12 | 0 | 0.14 | 0 |
| Norway Redfish | *Sebastes viviparus* | B |  | |  | 0.89 | 0.20 | 0 | 0 | 0 | 0 |
|  | *Sebastes spp. juv* |  |  | |  | 0.22 | 0.80 | 0 | 0 | 0.48 | 0 |
| Atlantic hookear sculpin | *Artediellus atlanticus* | MB |  | |  | 0.89 | 0.95 | 0.77 | 0.67 | 0.99 | 0.53 |
| Rough hookear sculpin | *Artediellus scaber* | A |  | |  | 0 | 0 | 0.12 | 0.03 | 0 | 0 |
| Arctic staghorn sculpin | *Gymnocanthus tricuspis* | MA |  | |  | 0 | 0.07 | 0.19 | 1 | 0.10 | 0 |
| Twohorn sculpin | *Icelus bicornis^2^* | MA |  | |  |  |  |  |  |  |  |
| Spatulate sculpin | *Icelus spatula^2^* | AB |  | |  |  |  |  |  |  |  |
|  | *Icelus* spp. |  |  | |  | 0 | 0.11 | 0.08 | 0.92 | 0.68 | 0.32 |
| Shorthorn sculpin | *Myoxocephalus scorpius* | MB |  | |  | 0 | 0.02 | 0.23 | 0.33 | 0.02 | 0 |
| Moustache sculpin | *Triglops murrayi* | B |  | |  | 1 | 0.56 | 0.62 | 0.42 | 0.45 | 0 |
| Bigeye sculpin | *Triglops nybelini* | A |  | |  | 0 | 0.17 | 0 | 0.25 | 0.98 | 1 |
| Ribbed sculpin | *Triglops pingelii* | AB |  | |  | 0 | 0.16 | 0.08 | 1 | 0.31 | 0.05 |
| Hooknose | *Agonus cataphractus* | B |  | |  | 0 | 0.01 | 0.08 | 0 | 0 | 0 |
| Polar sculpin | *Cottunculus microps* | MA |  | |  | 0.11 | 0.61 | 0 | 0 | 0.42 | 0.05 |
| Atlantic poacher | *Leptagonus decagonus* | AB |  | |  | 0 | 0.64 | 0.31 | 0.97 | 0.98 | 0.74 |
| Northern alligatorfish | *Ulcina olrikii* | A |  | |  | 0 | 0 | 0.31 | 0.97 | 0.07 | 0 |
| Lumpsucker | *Cyclopterus lumpus* | MB |  | |  | 0.22 | 0.26 | 0.15 | 0.17 | 0.08 | 0 |
| Leatherfin lumpsucker | *Eumicrotremus derjugini* | A |  | |  | 0 | 0 | 0 | 0.22 | 0.06 | 0 |
| Atlantic spiny lumpsucker | *Eumicrotremus spinosus* | MA |  | |  | 0 | 0.07 | 0.12 | 0.19 | 0.26 | 0 |
|  | *Careproctus spp.^3^* |  |  | |  |  |  |  |  |  |  |
| Variegated snailfish | *Liparis bathyarcticus^3^* | MA |  | |  |  |  |  |  |  |  |
| Gelantinous snailfish | *Liparis fabricii^3^* | A |  | |  |  |  |  |  |  |  |
| Kelp snailfish | *Liparis tunicatus^3^* | A |  | |  |  |  |  |  |  |  |
| Black seasnail | *Paraliparis bathybius^3^* | A |  | |  |  |  |  |  |  |  |
| Threadfin seasnail | *Rhodichthys regina* | A |  | | d |  |  |  |  |  |  |
| Liparids |  |  |  | |  | 0 | 0.77 | 0.27 | 0.81 | 0.99 | 1 |
|  | *Gymnelus spp.^4^* |  |  | |  | 0 | 0.04 | 0.04 | 0.28 | 0.24 | 0 |
|  | *Lycenchelys kolthoffi^4^* | A |  | |  | 0 | 0.02 | 0 | 0 | 0.09 | 0.05 |
|  | *Lycenchelys muraena* | A |  | | d |  |  |  |  |  |  |
|  | *Lycodes adolfi* | A |  | | d |  |  |  |  |  |  |
| Greater eelpout | *Lycodes esmarkii^4^* | MB |  | |  | 0 | 0.24 | 0 | 0.03 | 0.07 | 0.05 |
|  | *Lycodes eudipleurostictus* |  |  | |  | 0 | 0.17 | 0 | 0 | 0.07 | 0 |
|  | *Lycodes frigidus* | A |  | | d |  |  |  |  |  |  |
|  | *Lycodes gracilis^4^* | MB |  | |  | 0.78 | 0.74 | 0.08 | 0.08 | 0.11 | 0 |
|  | *Lycodes luetkenii* | A |  | | d |  |  |  |  |  |  |
|  | *Lycodes paamiuti^4^* |  |  | |  | 0 | 0.01 | 0 | 0 | 0 | 0 |
| Pale eelpout | *Lycodes pallidus^4^* | A |  | |  | 0 | 0.19 | 0.12 | 0.22 | 0.76 | 0.58 |
| Arctic eelpout | *Lycodes reticulatus^4^* | A |  | |  | 0 | 0.05 | 0 | 0.39 | 0.63 | 0 |
| Threespot eelpout | *Lycodes rossi^4^* | A |  | |  | 0 | 0.21 | 0 | 0.11 | 0.59 | 0.05 |
|  | *Lycodes seminudus^4^* | A |  | |  | 0 | 0.08 | 0 | 0.08 | 0.53 | 0.32 |
|  | *Lycodes squamiventer^4^* | A |  | |  | 0 | 0.01 | 0 | 0 | 0.02 | 0.05 |
|  | *Lycodonus flagellicauda^4^* | A |  | |  | 0 | 0.01 | 0 | 0 | 0 | 0 |
| Stout eelblenny | *Anisarchus medius* | B |  | |  | 0 | 0.09 | 0 | 0.67 | 0.14 | 0.11 |
| Daubed shanny | *Leptoclinus maculatus* | MB |  | |  | 0 | 0.77 | 0.96 | 0.97 | 0.98 | 0.21 |
| Slender eelblenny | *Lumpenus fabricii* | MA |  | |  | 0 | 0.14 | 0.42 | 0.42 | 0.28 | 0 |
| Snakeblenny | *Lumpenus lampretaeformis* | MB |  | |  | 0.67 | 0.65 | 0.65 | 0.81 | 0.58 | 0.05 |
| Northern wolffish | *Anarhichas denticulatus* | MB |  | |  | 0 | 0.64 | 0.12 | 0.06 | 0.07 | 0 |
| Atlantic wolffish | *Anarhichas lupus* | MB |  | |  | 1 | 0.41 | 0.85 | 0.17 | 0.13 | 0 |
| Sandeels | *Ammodytes sp^5^* |  |  | |  | 0.11 | 0.11 | 0.42 | 0.14 | 0.01 | 0 |
| Spotted wolffish | *Anarhichas minor* | MB |  | |  | 0.44 | 0.68 | 0.73 | 0.25 | 0.39 | 0 |
| Megrim | *Lepidorhombus whiffiagonis* |  |  | |  | 0.11 | 0 | 0 | 0 | 0 | 0 |
| Norwegian topknot | *Phrynorhumbuss norvegicus* | B |  | |  | 0.22 | 0 | 0 | 0 | 0 | 0 |
| Witch | *Glyptocephalus cynoglossus* | MB |  | |  | 0.33 | 0.05 | 0 | 0 | 0 | 0 |
| Long rough dab | *Hippoglossoides platessoides* | MB |  | |  | 1 | 1 | 1 | 0.97 | 0.98 | 0.21 |
| Atlantic halibut | *Hippoglossus hippoglossus* | MB |  | |  | 0.78 | 0 | 0 | 0 | 0 | 0 |
| Dab | *Limanda limanda* | MB |  | |  | 0 | 0.02 | 0.27 | 0 | 0 | 0 |
| Arctic flounder | *Liopsetta glacialis* |  |  | | s |  |  |  |  |  |  |
| Lemon sole | *Microstomus kitt* | B |  | |  | 1 | 0.01 | 0 | 0 | 0 | 0 |
| European plaice | *Pleuronectes platessa* | MB |  | |  | 0.44 | 0.04 | 0.69 | 0 | 0 | 0 |
| Greenland halibut | *Reinhardtius hippoglossoides* | MA |  | |  | 0.11 | 0.83 | 0 | 0.17 | 0.76 | 0.74 |

^1^Mostly *Benthostema glaciale*. ^2^*Icelus spatula* and *Icelus bicornis* were pooled in all analyses, and occurrence is given for the pooled *Icelus* spp. ^3^ In the cluster and correspondence analyses *Liparis* and *Careproctus* records were pooled with specimens only identified or recorded as the family *Liparidae*. ^4^Norwegian data from 2004-2007 on *Zoarcidae* were removed when calculating grid cell means due to poor species identification. ^5^Most likely only *Ammotydes marinus*.
