# Supplementary figures and images for: Demersal Fish Assemblages and Spatial Diversity Patterns in the Arctic-Atlantic Transition Zone in the Barents Sea
Source: PLoS One. 2012 Apr 17;7(4):e34924. doi: 10.1371/journal.pone.0034924 (PMC3328492; doi:10.1371/journal.pone.0034924)

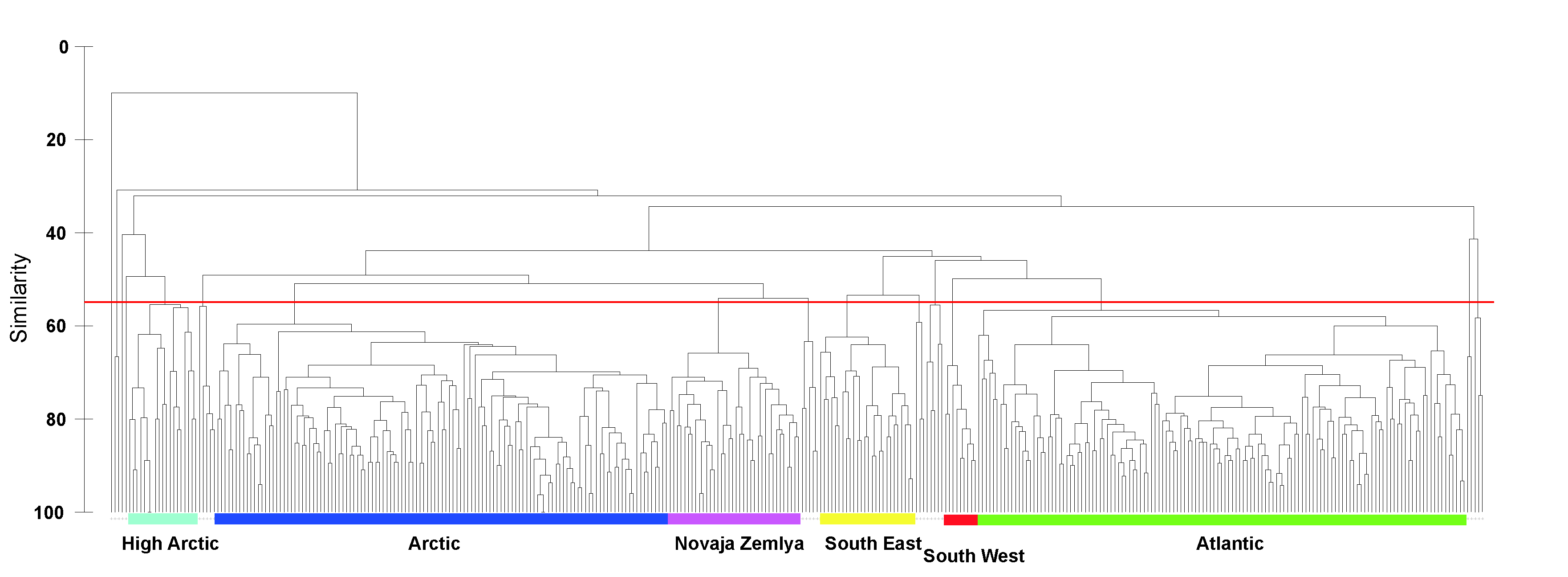

Supplement: Figure S1 — The full dendrogram from the hierarchical clustering on the grid cells (n = 374). The cut-off line at 55% similarity used to determine the clusters is shown in red. The main assemblages discussed in the text are identified by colored lines at the bottom of the dendrogram. (TIF) [file pone.0034924.s003.tif]
